# Editorial: Pathway, Genetic and Process Engineering of Microbes for Biopolymer Synthesis

**DOI:** 10.3389/fbioe.2020.618383

**Published:** 2020-12-23

**Authors:** Ignacio Poblete-Castro, Bruce A. Ramsay, Bernd H. A. Rehm

**Affiliations:** ^1^Biosystems Engineering Lab, Faculty of Life Sciences, Center for Bioinformatics and Integrative Biology, Universidad Andres Bello, Santiago, Chile; ^2^Chemical Engineering Department, Queen's University, Kingston, ON, Canada; ^3^Centre for Cell Factories and Biopolymers, Griffith Institute for Drug Discovery, Griffith University, Nathan, QLD, Australia

**Keywords:** polyhydroxyalkanoates, alginates, succinic and lactic acid, metabolic engineering, epothilone, poly(γ-glutamic acid) (γ-PGA), cell autolysis, (R)-3-hydroxy fatty acids

Together with the climate crisis, the heavy accumulation in oceans and soils of persistent pollutants, like polycyclic aromatics hydrocarbons and synthetic plastics, are the main drivers to impact nature and threaten human survival. It is particularly striking that most industrial polymers still originate from petrochemical sources—nearly 99% of the overall worldwide production. The result is materials that remain intact for centuries once deposited in the environment. Relying on non-renewable fossil chemicals limits our ability to establish a circular economy that promises to curb current emissions and contribute moderately to the global carbon cycle without surpassing its carrying capacity.

For decades, commercial biopolymers have also been produced by microbial fermentation since nature has endowed many bacteria from urban sites to extreme environments (Orellana-Saez et al., 2019) with the enzymatic machinery to assemble these macromolecules. Despite the rapid pace of innovation, microbial biopolymers are still expensive to synthesize because the generally oxygen-intensive fermentation processes, downstream processing, and carbon feedstock cost boost production expenses (Oliveira et al., 2020). The biopolymers must additionally possess specific mechanical and physical properties to be processed industrially into products with a variety of applications (Moradali and Rehm, 2020).

Beyond their initial use as raw materials for packaging and films, biopolymers are finding new applications in biomedicine serving as drug nanocarriers, wound dressings, and tissue engineering materials along with usages as pharmaceutical coatings. The most profitable biopolymers are the family of polypeptides. Particularly, polyketides (PKs) have lured special attention in the clinical sector as they are efficient anticancer drugs and exhibit biological activity (antibiotics) against resistant bacteria and fungi. The food industry also employs biopolymers as thickeners and stabilizer agents. Moreover, water treatment and remediation technologies benefit from biopolymer physicochemical features as they act as dye removers and flocculation agents. Combining bacterial genome engineering with optimized cultivation modes in bioreactor enables the biosynthesis of tailor-made macromolecules from renewable carbon substrates and waste streams that meet technical requirements essential for medical and environmental applications. Together these strategies promise to accelerate the design of cost-effective processes that can compete against the classical petrochemical route.

This special issue sought to gather original studies and review papers covering the rational engineering of the gene repertory of microbes, synthetic bacterial consortia, and bioprocess optimization for efficient biopolymer production. Researchers across the globe poured efforts into the microbial synthesis of poly(3-hydroxyalkanoates) (PHAs), poly(γ-glutamic acid) (γ-PGA), epothilone, extracellular hydroxyalkanoic acids, alginates, and the starting building blocks succinate and D-lactate.

Two research groups studied bacteria of the *Bacillus* genus specialized in producing the versatile polyamide, poly(γ-glutamic acid). Wang et al. expanded the range of molecular weights (6.82·10^4^ to 1.78·10^6^ Da) of the biosynthesized γ-PGA by modulating the expression of the poly(γ-glutamic acid) depolymerase in *B. licheniformis* via promoter and signal peptide engineering. In another study, Halmschlag et al. investigated the metabolome profile of *B. subtilis* with altered rates of γ-PGA synthesis, using different promoters and induction degrees. They showed that if glutamate was not added to the culture, *B. subtilis* increased the phosphoenolpyruvate concentration while producing high levels of the biopolymer. They postulated that a reduction of the growth rate and diverting carbon fluxes toward glutamine synthesis could enhance γ-PGA production in *B*. *subtilis*.

The bacteria-derived anticancer compound epothilone is one of the most effective drugs against breast cancer with tolerable side effects. To improve epothilone synthesis in *Sorangium cellulosum*, Ye et al. targeted suitable promoters for overexpressing epothilone biosynthetic genes. This strategy yielded high-expression of the *epoA*, epoC, and epoK genes by inducing the P3 promoter contained in both a TALE-VP64 and dCas9-VP64 vectors, reaching elevated amounts of epothilone B and D.

Many articles evolved around PHAs synthesis. First, Velázquez-Sánchez et al. reviewed the importance of altering the regulatory circuits governing PHA accumulation in well-established host producers for modulating physicochemical properties and biopolymer yields. Similarly, Godard et al. applied a multi-omic approach to decipher the molecular mechanism behind the osmotic-stress adaptation of *Bacillus megaterium* that triggers enhanced synthesis of poly(3-hydroxybutyrate) (PHB). NADPH's oversupply, accompanied by an altered abundance of enzymes and genes belonging to central carbon metabolism, increased acetyl-CoA flux toward PHB synthesis. Engineering the industrial host *Escherichia coli*, Li et al. successfully produced high levels of PHB optimizing the ribosome binding site sequences of several genes attaining 85% of the cell dry mass (CDM) as PHB. Process optimization is another means to increase biopolymer productivity. Oliveira-Filho et al. utilized xylose, a promising 5-carbon sugar derived from hemicellulose, for fed-batch PHB production in *Burkholderia sacchari* under nitrogen or phosphorus limitation, resulting in both cases in a yield of 0.37 (gPHB ${\text{g}}_{{\text{xylose}}^{-1}}$).

Novel strategies for downstream processing of intracellular biopolymers are essential for decreasing the production costs. Delving into this topic, Yañez et al. present the latest advances of metabolically engineered strains and process setup for secreting *R*-3-hydroxy fatty acids and cell disruption for PHA release. On the same subject, an original study by Poblete-Castro et al. rationally engineered *Pseudomonas putida*, a native medium-chain length PHA producer, to alter the cell's osmotic state overexpressing the major porin enzymes along with inactivating the inner membrane enzyme MscL. When the engineered *P. putida* was given an osmotic upshift and then a rapid passage to a hypotonic condition, the cells suffered membrane disruption (95% of the population) after 3 h and displayed a PHA recovery of 94%.

Synthetic bacterial consortia are sustainable paths to produce PHAs from low-cost feedstocks. Taking advantage of the CO_2_ conversion ability of *Synechococcus elongatus*, Hobmeier et al. grew this cyanobacterium autotrophically to secrete sucrose in a first stage and subsequently form PHA in Δ*nasT P. putida*, unable to consume nitrate and harnessed with genes necessary for sucrose catabolism, finally amassing 14.8% of CDM as PHA from CO_2_.

Another important microbial biopolymer is alginate, which finds uses in the pharmaceutical and food industry. Constructing a transposon insertion mutant library, Mærk et al. screened for regulatory and metabolic genes involved in impaired alginate production in *Azotobacter vinelandii*. Genes belonging to the peptidoglycan, the TCA cycle, and vitamin biosynthesis appear to be crucial for assembling these macromolecules, while the addition of succinate or lysine improved alginate yields.

Finally, two engineered *E. coli* strains, each specialized in the degradation of xylose and glucose, produced the bioplastic precursor monomers succinate and D-lactate simultaneously. Flores et al. demonstrated that by co-culturing these cell factories in a defined population ratio, the bacterial community produced 88 g L^−1^ D-lactate and 84 g L^−1^ succinate.

Together, the studies published in this Research Topic reveal the ongoing efforts to obtain tailored biopolymers for various applications via fine-tuning gene expression and the host strains' pathway operation. Many challenges remain, especially in attaining maximal biopolymer production performance of engineered cell factories during fermentation in bioreactors with the desired monomer composition and physical properties.

## Author Contributions

IP-C conceived the idea of the Research Topic and served as editor. BAR and BHRA served as editor. All author contributed to the editorial and approved the submitted version.

## Conflict of Interest

BAR holds a patent for a method of synthesizing medium chain length polyhydroxyalkanoate. The remaining authors declare that the research was conducted in the absence of any commercial or financial relationships that could be construed as a potential conflict of interest.
